# Applications of Next Generation Sequencing to the Analysis of Familial Breast/Ovarian Cancer

**DOI:** 10.3390/ht9010001

**Published:** 2020-01-10

**Authors:** Veronica Zelli, Chiara Compagnoni, Katia Cannita, Roberta Capelli, Carlo Capalbo, Mauro Di Vito Nolfi, Edoardo Alesse, Francesca Zazzeroni, Alessandra Tessitore

**Affiliations:** 1 Department of Biotechnological and Applied Clinical Sciences, University of L’Aquila, Via Vetoio, Coppito 2, 67100 L’Aquila, Italy; veronica.zelli@univaq.it (V.Z.); chiara.compagnoni@graduate.univaq.it (C.C.); roberta.capelli@graduate.univaq.it (R.C.); mauro.divitonolfi@graduate.univaq.it (M.D.V.N.); edoardo.alesse@univaq.it (E.A.); francesca.zazzeroni@univaq.it (F.Z.); 2 Center for Molecular Diagnostics and Advanced Therapies, University of L’Aquila, Via Petrini, 67100 L’Aquila, Italy; 3 Medical Oncology Unit, St Salvatore Hospital, Via L. Natali 1, 67100 L’Aquila, Italy; kcannita@gmail.com; 4 Department of Molecular Medicine, University of Rome “La Sapienza”, Viale Regina Elena 324, 00161 Rome, Italy; carlo.capalbo@uniroma1.it

**Keywords:** next generation sequencing (NGS), whole exome sequencing (WES), whole genome sequencing (WGS), hereditary tumors, familial breast/ovarian cancer, BRCA1, BRCA2

## Abstract

Next generation sequencing (NGS) provides a powerful tool in the field of medical genetics, allowing one to perform multi-gene analysis and to sequence entire exomes (WES), transcriptomes or genomes (WGS). The generated high-throughput data are particularly suitable for enhancing the understanding of the genetic bases of complex, multi-gene diseases, such as cancer. Among the various types of tumors, those with a familial predisposition are of great interest for the isolation of novel genes or gene variants, detectable at the germline level and involved in cancer pathogenesis. The identification of novel genetic factors would have great translational value, helping clinicians in defining risk and prevention strategies. In this regard, it is known that the majority of breast/ovarian cases with familial predisposition, lacking variants in the highly penetrant BRCA1 and BRCA2 genes (non-BRCA), remains unexplained, although several less penetrant genes (e.g., ATM, PALB2) have been identified. In this scenario, NGS technologies offer a powerful tool for the discovery of novel factors involved in familial breast/ovarian cancer. In this review, we summarize and discuss the state of the art applications of NGS gene panels, WES and WGS in the context of familial breast/ovarian cancer.

## 1. Introduction

Inherited genetic defective variants significantly contribute to familial cancers. In the beginning, the study of hereditary cancer was based on the linkage analysis of numerous pedigrees, which led, in 1994, to the isolation of BRCA1 and BRCA2, the main predisposing genes for hereditary breast/ovarian cancer (BC/OC) syndrome [1,2]. Later, other genetic risk factors for BC/OC were identified as well. The extensive use of Sanger DNA sequencing, often associated with upstream prescreening techniques (e.g., single stranded conformational polymorphism or DHPLC) [3], allowed the detection and the characterization of germ-line gene mutations responsible for cancer susceptibility. However, the above-mentioned technologies analyze just one gene, or its parts, at a time, making the procedures time-consuming. In the first decade of the 21st century, after the human genome project completion, a significant step forward was provided by the creation of next generation sequencing (NGS) technologies, able to perform multi-gene analysis or sequencing of entire exomes, corresponding to the DNA protein-coding regions, transcriptomes, including coding and/or non-coding RNA transcripts, or genomes, consisting of both exon and non-coding intron DNA regions [4,5].

This review is focused on describing the applications and potentiality of NGS technologies for the detection of variants in genes already known as responsible for familial breast and ovarian cancer, and novel susceptibility genes/variants as well.

## 2. Breast and Ovarian Cancer

Breast cancer (BC) is the most diagnosed cancer in women worldwide. In 2018, its incidence was estimated at more than 2 million new cases. BC accounts for about 11.6% of all cancer cases and for 6.6% of all cancer-related deaths [6]. On the other hand, ovarian cancer (OC) is less frequent, accounting for about 1.6% of all tumor cases and 1.9% of cancer-related deaths, but is more lethal compared to BC [6]. Approximately 90%–95% of breast tumors and 80% of ovarian tumors are sporadic, whereas the remaining 5%–10% and 20%, respectively, are classified as familial or hereditary. In the latter group, only 20%–25% of BC/OC cases are due to inheritance of the highly penetrant cancer susceptibility genes BRCA1 and BRCA2, involved in DNA repair mechanisms [7]. The prevalence of BRCA1/2 alterations varies across ethnic groups and geographical areas, with higher frequencies observed in white populations from Europe and Australia (17.6%–29.8%) and lower frequencies recorded in Asian countries (9.4%–21.7%) [8].

Thus, a high fraction of cases with familial predisposition (non-BRCA) lack mutations in those highly penetrant genes and remain unexplainable [9].

It has been hypothesized that other moderately-penetrant genes, coding for proteins that interact with BRCA1/2 or act in the same DNA repair pathway, would be likely candidates for hereditary BC/OC susceptibility. As expected, genes such as ATM, CHEK2, PALB2, BRIP1 and RAD50 have shown to play a role in familial/hereditary forms of BC/OC [7,10,11]. Compared with highly penetrant genes, for which inherited mutations confer a five-fold or greater risk, mutations in those moderately-penetrant genes are associated with a two- to four-fold risk increase. Furthermore, genetic testing for specific cancer susceptibility genes, such as TP53, PTEN, CDH1 has also proven to be indicated in selected BC patients as well [11].

Although mutations in several genes have been found to predispose to BC/OC, these account for only a small fraction of cases and most potential hereditary cases still remain unexplained [12]. Thus, it is likely that additional gene mutations remain unidentified, especially in the category of moderate- to low-penetrant gene variants.

In this context, a polygenic risk model, in which the combination of many common single nucleotide polymorphisms (SNPs) may contribute to increase the BC/OC risk, has been suggested [13]. These SNPs act as common low-penetrance variants, that individually confer a small increased risk but, in combination, confer a much larger risk in the population. This hypothesis has been confirmed by several genome-wide association studies (GWAS) that identified more than 70 SNPs associated with BC [14,15,16,17,18,19,20] and more than 30 SNPs associated with OC [21,22,23]. Taken together, these data suggest that the identification of additional susceptibility genes or gene variants is crucial to better define the large fraction of BC/OCs due to genetic predisposition but lacking pathogenic variants in highly-penetrant BRCA1 and BRCA2 or other less-penetrant genes, with important clinical implications in terms of risk assessment and personalized approach to prevention and treatment.

## 3. NGS Technologies

Next-generation sequencing (NGS) technology has revolutionized the clinical approach to genetic testing across many areas of medicine, such as oncology. The great power of NGS technology is the capability to massively sequence millions of DNA reads, allowing for an accurate characterization of the “status” of multiple genes, by using very low amount of nucleic acids with considerable time and cost reduction. Several second and third generation platforms, showing high performance based on different chemical/physical principles and ability to respond to different experimental needs, are currently commercially available [24]. Thanks to its ability to detect high numbers of variants simultaneously, NGS technology has been widely used in many studies in order to provide insight into the characterization of the tumorigenic process and tumor heterogeneity [25,26]. The analysis of the molecular landscape of tumors can provide information of clinical utility in terms of diagnosis, prognosis and therapy response prediction. Furthermore, NGS technology provides a very powerful method for the identification and discovery of novel genes responsible for cancer susceptibility, with the possibility of counseling patients and their families regarding screening, surveillance, and risk-reducing options [27]. During the last decade, the advent of NGS made it possible to obtain not only targeted sequencing of several genes, such as those highly or less penetrant already known to be involved in BC/OC susceptibility, but also sequencing of entire exomes, transcriptomes or genomes for the identification of novel genes or gene variants putatively responsible for BC/OC predisposition.

The “easiest” NGS approach is “targeted gene sequencing”, which allows the analysis of selected genes or specific subsets of gene regions, whose involvement in specific diseases has already been observed or suggested. NGS gene-panels are widely used, since they represent a very high-throughput and cost-effective screening method for sequencing of specific targets of interest; they offer the possibility of massive parallel multigene analysis in few days, with significant time and cost reduction and by using a very low amount of nucleic acid [28].

Whole exome sequencing (WES), where DNA coding regions are captured and sequenced at a deep level, has proven to be an effective procedure for detecting disease-causing variants and discovering new target genes. Compared to targeted gene sequencing, WES allows one to enhance sequencing power, providing a more complete investigation of the genomic landscape. Currently, WES is the most used NGS technique for the identification of rare genetic variants associated with disease [29,30]. However, WES analysis provides information about exons, confining the analysis to the coding regions of the genome. Therefore, one of the most important limitation of this approach is the omission of variants in non-coding regulatory regions that, in some cases, have been implicated as cancer driver mutations [31,32]. An example is represented by both germline and somatic TERT promoter mutations that lead to the increase of telomerase activity and so promote the immortalization of cancer cells [33]. Particularly, germline TERT promoter mutations have been associated with familial cancer risk [34].

In this regard, whole-genome sequencing (WGS), an approach based on the sequencing of the entire genome, provides the most complete analysis for the characterization of the genomic profile and the possible biological consequences, leading to the discovery of new molecular alterations in coding as well as non-coding regions [35]. Compared with WES, WGS is more expensive and requires a greater amount of starting material. Furthermore, despite the great potential of WGS analysis, it shows several difficulties, mainly related to the high amount of generated data and their validation and interpretation [36].

Overall, despite the enormous potential of WES and, mostly, WGS, data management, data analysis and biological interpretation are critical to achieve optimal results [37,38]. NGS sequencing produces millions/billions of massive sequencing reads; thus, a huge amount of raw data is generated, especially when high sequencing depth is required. Therefore, one of the most relevant challenges is how to manage the enormous amount of data and how to choose the best computational methods and tools for analysis, taking into consideration, for both approaches, the lack of a standardized data analysis method and the different levels of performance shown from different tools employed in the NGS workflow [28].

To date, comprehensive multi-gene panels, mainly focused on genes involved in DNA repair pathways, have been extensively used in BC/OC research and clinical application [39]. In addition, more exhaustive examination has been performed by whole exome [40] or genome sequencing studies. In the next sections, the state of the applications of the gene panel, WES and WGS approaches will be discussed in the context of familial BC/OC. The studies here reported are summarized in Table 1.

## 4. NGS Applications for BRCA1/2 Gene Analysis

Due to the large size of BRCA1 and BRCA2, and to the varied nature of the mutations scattered across the whole length of both genes, several tests, often labor intensive, were conceived and used. The protein truncation test [59], an indirect method for mutation analysis, was able to detect the presence of truncating variants but missed those missense and structural gene alterations. The “gold standard” Sanger sequencing was considered the best approach to identify point mutations, small deletions or insertions, but it is time consuming and expensive. Other mutation analysis methods, based on screening by DHPLC [60] followed by direct sequencing, have been employed. Moreover, multiplex ligation dependent probe amplification (MLPA) was used to detect large genomic rearrangements [61]. The advent of NGS technologies made it possible to perform multi-gene massive sequencing, with very sensitive detection of gene variants, even complex, and copy number variations as well [62,63]. Several works [64,65,66,67] demonstrated the advantage of using NGS with respect to traditional methods for BRCA variant identification in terms of sensitivity, cost and time reduction. Importantly, the latter is very relevant for therapy decision-making, in consideration of the possible use of anti-PARP drugs for ovarian and breast cancer treatment [68,69,70]. In 2012, two studies developed a NGS method, based on long-range PCR of BRCA1/2 exons and flanking regions [71] or of the entire genomic regions [72], which allowed identification of 100% of the BRCA1/2 predisposing mutations, as well as other genomic variations in a cohort of patients with a family history for BC/OC, already screened for BRCA1/2 mutations by denaturing HPLC and/or Sanger sequencing. Later, Hernan et al. [73] and Hirotsu et al. [74] identified two novel frameshift mutations in BRCA2 and one mutation in BRCA1, plus two mutations in BRCA2, respectively, by using commercially available kits for NGS, further confirming the usefulness of this technology in clinical molecular diagnostics. In the same way, Kluska et al. [75] identified a wide spectrum of mutations, some of them recurrent, in 512 Polish women with familial or early onset BC/OC. Jouali et al. [76] analyzed 15 patients from 68 Moroccan families with BC/OC predisposition and found several BRCA mutations, including one novel frameshift in BRCA1. Twenty-nine novel BRCA1/2 gene variants were identified by Santonocito et al. [77] in 1400 consecutive Caucasian patients with a BC/OC family history, further confirming the power of this technology in BRCA genotyping with the aim of better managing patients and relatives with a genetic predisposition. In a recent study [78], the authors performed exome sequencing-based screening in an unselected research cohort of adult volunteers in order to identify pathogenic and likely pathogenic variants in BRCA1/2 genes. Results of this analysis highlighted the power of population screening in the identification of a higher proportion of BRCA1/2 mutation carriers compared to current criteria for genetic testing, with relevant implications and opportunities in terms of prevention strategies.

Some reports [66,79,80] described simultaneous NGS sequencing data collection and identification of BRCA rearrangements, simply by evaluating the presence of copy number variations (CNVs). This provides a fast and useful global first-step analysis that leads to more in-depth confirmatory investigation on positive samples by complementary tests, such as MLPA. Commercially available diagnostics kits currently take advantage of this NGS potentiality.

As known, the expected frequency of germline BRCA mutations is 50%. Interestingly, Friedman et al. [81] highlighted a constitutional low-level *de novo* mosaicism, identifying by NGS a pathogenic BRCA1 mutation (c.1953dupG, 5% of reads) in DNA extracted from BUCCAL SWAB, leucocytes, and normal breast tissues obtained from a patient affected by early-onset, triple negative breast cancer, who showed the BRCA1 mutation in tumor tissue (approximately 50% of reads). The mutation was missed in germline DNA by conventional Sanger sequencing, whose detection limit is known to be approximately 15%–25% [82,83], highlighting the utility of NGS in the identification of mosaicism events that are not always detectable by traditional sequencing methods, due to low mutation frequency.

## 5. Gene-Panel Sequencing Approach in Familial Non-BRCA Breast/Ovarian Cancer

In order to identify other BC/OC susceptibility genes/variants, especially in familial non-BRCA patients, panels for analysis of cancer-related genes other than BRCA1/2 were used as well. In a prospective study of Tung et al. [41], a panel of 25 genes was used to test 488 patients with stage I to III breast cancer with or without family history. Several germline mutations in genes related to breast or other types of cancer were found, indicating the need to identify new predisposing factors responsible for non-BRCA cancers. Byers et al. [42] took advantage of NGS for the analysis of a 10 genes’ panel, associated with BRCA1/2 RNA sequencing, in breast/ovarian-male breast cancer families with no identified pathogenic exon variants and a copy number analysis of BRCA1/2. The study showed little contribution of RNA sequencing and NGS gene panel testing, further highlighting the need to identify other high-risk genes for patients with a familial history.

A panel of 94 genes involved in hereditary tumors was used to assess the presence of germline mutations in 255 women with a familial history. Pathogenic BRCA1/2 mutations were found in 57 patients, whereas 17 showed pathogenic variants in other less-penetrant genes (e.g., PALB2, ATM, BRIP1, RAD51D, MSH6, PPM1D, RECQL4, ERCC3, TSC2, SLX4). Based on the clinical characteristics of the latter group, mutations in genes other than BRCA1/2 seemed to confer a high risk of cancer development, indicating that a wider gene analysis could improve protocols of surveillance [43]. A very recent work by Suszynska et al. [44] showed the results of a large meta-analysis based on results obtained from multi-gene panel testing. After evaluating 37 genes usually analyzed in BC/OC predisposition, the authors highlighted several non-BRCA genes associated with higher breast/ovarian cancer risk, providing evidence on the possibility of identifying groups of genes more specifically connected to BC/OC. Among them, CDKN2A showed a contribution to breast cancer risk that was comparable to that conferred by BRCA2, whereas RAD51C, RAD51D, BRIP1 were proven to be responsible for an increase in ovarian cancer risk. This work points out the great heterogeneity shown by the gene panel-based studies in terms of number of cases and selection strategies, analyzed genes and data elaboration, suggesting more standardized workflows are needed among molecular genetics laboratories.

## 6. WES Approach in Familial Non-BRCA Breast/Ovarian Cancer

In addition to multi-gene panels, including genes already known to be involved in hereditary cancers, several studies have aimed at identifying novel factors putatively responsible for familial BC/OC. In this context, NGS offers high performance, allowing one to sequence entire exomes, genomes and transcriptomes in a fast and cost-effective manner. Whole-exome sequencing (WES) is a technology able to provide information about almost all the protein-coding DNA sequences. For this reason, the WES approach is thought to be particularly suitable in cancer genetics for the isolation of new putative genes playing a role in non-BRCA patients with familial susceptibility, which could, in addition, potentially aid in defining risk stratification. In 2012, Park et al. [45] identified by WES the gene XRCC2, a RAD51 paralog, as putatively correlated to BC risk increase. In the same year, Thompson et al. [46] analyzed 33 patients with a family history by WES and described FANCC and BLM, responsible for the autosomal recessive disorders Fanconi Anemia and Bloom Syndrome, as BC susceptibility genes. The same technology was used by Kiinski et al. [47] to identify FANCM, with the presence of a nonsense mutation c.5101C>T, as a BC susceptibility gene in Finnish families. Park et al. [48] identified RINT1, originally described as coding for a RAD50 interacting protein, as correlated with an intermediate level BC risk. Cybulski et al. [49] analyzed 144 Polish and 51 French-Canadian women with BC, negative for BRCA1/2, CHEK2, NBN (NBS1), and PALB2 founder mutations, selected based on their family history and/or young age at the onset. The authors identified rare and recurrent variants of RECQL, a gene involved in preventing double-stranded DNA breaks. The same gene was also identified as potentially associated with BC by Sun et al. [50], who analyzed the exomes of Chinese non-BRCA patients. Maatta et al. [51] performed exome sequencing of 13 non-BRCA high-risk Finnish families. After filtering, 18 candidate variants in the DNA damage-response (DDR) pathway were identified and further validated by conventional methods in cohorts of BC/OC female patients, female controls and breast tumors. Results showed that enrichment of multiple defects in DDR genes are related to BC predisposition in those high-risk families, opening the possibility of further studies. Tavera-Tapia et al. [52] analyzed the exome of a non-BRCA Spanish family and found a novel germline ATM mutation (c.5441delT; p.Leu1814Trpfs*14). In the same study, the ATM gene was further analyzed in a cohort of 392 non-BRCA cancer families and showed 1.78% prevalence of mutations in non-BRCA familial BC/OC and a 1.94% frequency in BC, suggesting that testing of this gene in Spanish non-BRCA families should occur. Hamdi et al. [53] performed whole exome sequencing in non-BRCA Tunisian families and identified twelve relevant high-risk variants and four new BC candidate genes (MMS19, DNAH3, POLK, KATB6). In a large study by Lu et al. [54], more than 11,000 patients with clinical features of breast and/or ovarian cancer and almost 4000 controls were subjected to exome-sequencing to analyze the BC/OC features in depth, to confirm the involvement of known genes other than BRCA, and to identify new genes potentially associated with the disease. The study produced a huge amount of data, in terms of genes and pathogenic variants. After filtering, enrichment of pathogenic variants was identified in four non-BRCA genes, which were related to BC risk (ATM, CHEK2, PALB2, and MSH6). On the other hand, increased risk for OC was linked to MSH6, RAD51C, TP53, and ATM. Genes belonging to the MRN complex (RAD50, MRE11, NBN-NBS1) and CDKN2A were not correlated with increased BC/OC risk. In addition, no association was shown between BC and OC susceptibility BRIP1, RAD51C, RAD51D, MSH2, and PMS2 genes, partly confirming results of other studies. Overall, the study highlighted the need to (i) standardize NGS procedures for obtaining high-quality results and (ii) analyze pathological samples and controls with well characterized clinical data. Moreover, extending the analysis was suggested, not only to protein truncating and known pathogenic mutations, but also to a wider spectrum of variants, especially in less-studied genes that could be putatively involved in BC/OC susceptibility. Another study [55] examined 113 DNA repair genes, filtered from whole exome sequencing data of a well characterized and homogeneous group of familial BCs. Other than PALB2, ATM, and CHEK2 deleterious-predicted variants, the authors found, for the first time, BC susceptibility associated with FANCI, MAST1, POLH and RTEL1 gene mutations. Weitzel et al. [56] analyzed a large cohort of more than 1000 non-BRCA Hispanic women by exome sequencing, 4.5% of whom carried pathogenic variants in cancer susceptibility genes (CHEK2, PALB2, ATM, TP53, BRIP1, CHD1, and NF1). Among them, the most frequent were reported in PALB2 and CHEK2, further confirming their involvement in some non-BRCA cancers. Whole exome sequencing was also applied to 52 individuals from 17 Greek families, in which at least one patient was negative for known hereditary BC risk variants [57]. Pathogenic variants were found in already-described genes (BARD1, encoding a ligase interacting with BRCA1; MEN1, involved in multiple endocrine neoplasia syndrome) and a workflow was used to identify novel variants outside the known risk genes: rare loss-of-function variants were detected in MDM1, encoding a nuclear protein, and the NBEAL1 gene, playing a role in molecular mechanisms including vesicular transport, apoptosis and receptor signaling. A missense variant in SETBP1, coding for a protein that binds to the nuclear oncogene SET, and C7orf34, with predicted damaging effects, was detected as well.

Despite the more than a dozen WES-based studies reported here and the comparable, in terms of order of magnitude, number of interesting genes identified, with most involved in tumor key mechanisms, no unequivocal genes, specifically responsible for non-BRCA BC/OC cases, were isolated. Nevertheless, these analyses provided significant and extensive deepening of knowledge of the complex landscape of familial breast and ovarian cancers. In the future, one could expect that selected, homogeneous groups of patients, with adequate size, and novel bioinformatics analysis pipelines for results’ filtering and interpretation could enhance the list of putative susceptibility genes/variants to be further validated.

## 7. WGS Approach in Familial Breast/Ovarian Cancer

Other than exome sequencing, whole genome sequencing (WGS) has gained attention due its power in analyzing both coding and non-coding regions. WGS produces high-throughput data, with the consequent need for powerful and focused bioinformatics analysis. A work by Nones et al. [58] compared 78 paired germline and tumor DNA samples obtained from women carrying BRCA1 or BRCA2 pathogenic mutations, and from non-BRCA patients. Matched analysis allowed the authors to confirm biallelic inactivation of genes, playing a role in cancer risk increase, which lead to the accumulation of somatic mutations. Loss of function of BRCA1/2 and PALB2 was correlated with mutation burden and defective homologous recombination (HR). Thirteen non-BRCA tumors were BRCA-proficient and showed structural rearrangements correlated with oncogene amplification and germline pathogenic variants at the level of TP53, ATM and CHEK2. In conclusion, this study highlighted, for the first time, the importance of the whole genome sequencing-based approach, focused on the analysis of paired germline-tumor DNA, to shed light on important mechanisms of genomic instability underlying familial breast cancer.

## 8. Pros and Cons of NGS Approaches for the Analysis of Familial Breast/Ovarian Cancer

Although NGS technologies have a well-established potential in BC/OC clinical diagnostics, as well as molecular research, there are still many challenges associated, for example, with the accurate determination of large genomic rearrangements, the interpretation of variants of unknown significance in known susceptibility genes and the interpretation of pathogenic variants in genes not previously associated with BC/OC genetic risk.

Conventional methods, including Sanger sequencing and MLPA, are time-consuming and very expensive, particularly for the analysis of large genes such as BRCA1 and BRCA2. In this context, NGS technologies offer a powerful alternative, improving the speed and the efficiency of molecular testing [67]. Furthermore, it was demonstrated that NGS is more sensitive for detecting BRCA1/2 sequence variants compared to previous techniques such as PTT, SSCP and DHPLC, and, for this reason, there is often the need to re-analyze patients by using NGS technologies, due to the false negative results obtained by using the above mentioned screening methods [84]. Concerning large genomic alterations, even though studies are under development, detection of CNVs by NGS has not yet been fully validated in clinical diagnostics, and improvement of enrichment methods and bioinformatics analysis processes are necessary to allow the application of NGS as a routine method for the detection of BRCA1/2 CNVs analysis [85].

Over the last few years, thanks to the extensive use of NGS technology, several genes other than BRCA1/2, have been associated with increased BC/OC risk. To date, a wide range of NGS panels are available for the analysis of hereditary BC/OC [86]; generally, these panels include high-penetrance BC/OC genes (BRCA1 and BRCA2), moderate/low-penetrance genes (e.g., PALB2, CHEK2 and ATM), mismatch repair genes (e.g., MLH1 and MSH2), and genes related to hereditary cancer syndromes (e.g., CDH1, PTEN, STK11 and TP53) [87]. Overall, a critical factor for NGS panel use is that a high number of variants of unknown significance (VUS) are detected, for which clinical management is unclear.

To date, over 35 genes have been suggested as possible BC susceptibility genes, however a statistically significant association with BC risk has been established only for a minor fraction of them [40]. This result highlights the need to identify additional BC/OC susceptibility genes able to explain the high fraction of hereditary cases not attributable to mutations in known BC/OC predisposing genes.

In this context, WES and WGS analyses offer a great opportunity to discover novel genes involved in BC/OC susceptibility. However, the success of this type of study relies on many factors, including size and selection of patients recruited and data analysis strategies as well. Adequate validation experiments in independent series and case-control analyses are also essential to obtain information of clinical utility. For some putative novel BC/OC associated genes, identified by WES studies, further independent investigations and case-control analyses have not been performed and thus the associated BC/OC risk is unknown [40]. Furthermore, it is important to note that, in some studies, BC cases that were exome-sequenced had no mutation reported [40].

Overall, well-designed studies that include well selected patients/families, adequate sample size (due to the rarity of variants), homogeneity of the population analyzed, validation experiments in independent cohorts and case-control studies to assess the risk, are essential to improve the success of WES studies and the probability of discovering new BC/OC-associated genes.

## 9. Conclusions

The recent advances in high-throughput technologies and the advent of second and third generation sequencing methods have shown great potential for medical genetic research with translational value, providing a significant improvement in terms of genetics understanding. Several studies led to the identification of novel susceptibility genes/variants associated with familial BC/OC, analysis of which can help clinicians assess risk stratification and prevention strategies. The NGS-based studies here reported did not always analyze univocal genes other than BRCA1 and BRCA2, but one could expect that further studies, focused on analysis of well selected patients/families and coupled to the improvement and standardization of bioinformatics investigations, will have a strong chance of identifying novel genes/variants involved in familial breast and ovarian cancers. Ethnicity, number of analyzed cases necessary to identify rare variants, and case/control studies for risk assessment should also be considered. Moreover, these technologies can provide a powerful tool for understanding additional mechanisms operating in familial cancer that can be revealed by the wide comparison, now possible, of matched germline and tumor DNA.

## Figures and Tables

**Table 1:** NGS studies in familial breast/ovarian cancer.

Type of Study	Reference	NGS Approach	Country or Ethnicity	Samples/Groups Analyzed	Most Relevant Genes Emerged	Validation/Additional Analysis
Targeted sequencing studies	Tung et al., 2016 [41]	Targeted sequencing (*ATM, BARD1, BRIP1, CDH1, CHEK2, NBN, PALB2, PTEN, STK11, TP53, APC, BMPR1A, CDK4, CDKN2A, EPCAM; MLH1, MSH2, MSH6, MUTYH, PMS2, RAD51C, RAD51D, SMAD4*)	USA	488 BC patients with or without BC/OC family history	*BRCA1*, *BRCA2*, *CHEK2*, *ATM*, *BRIP1*, *PALB2*, *PTEN*, *NBN*, *RAD51C*, *RAD51D*, *MSH6 and PMS2*	
	Byers et al., 2016 [42]	Targeted sequencing (TP53, CDH1, STK11, PTEN, PALB2, BRIP1, RAD51C, RAD51D, ATM and CHEK2)	UK	42 individuals from 45 high-risk BC/OC-male BC families, negative for BRCA1/2	*RAD51D*, *ATM*, *CHEK2*	
	Tedaldi et al., 2017 [43]	Targeted sequencing (panel of 94 genes involved in hereditary tumors)	Italy	255 HBOC patients	*BRCA1*, *BRCA2*, *PALB2*, *ATM*, *BRIP1*, *RAD51D*, *MSH6*, *PPM1D*, *RECQL4*, *ERCC3*, *TSC2*, *SLX4*	
	Suszynska et al., 2019 [44]	Meta-analysis of 48 targeted-sequencing studies (37 genes evaluated)	More Countries	about 120,000 BC/OC patients and 120.000 controls	*BRCA1/2*, *CDKN2A*, *PTEN*, *PALB2*, *TP53* in Breast cancer; *BRCA1/2*, *RAD51D*, *PTEN*, *TP53*, *BRIP1*, *RAD51C*, *MSH6*, *MSH2* in Ovarian cancer	
WES studies	Park et al., 2012 [45]	WES	More Countries	13 BC families	*XRCC2*	Case-control analysis in 1308 early-onset BC cases, 689 multiple-case BC families and 1120 healthy controls
	Thompson et al., 2012 [46]	WES	Australia	33 individuals from 15 BC families, negative for BRCA1/2	*FANCC* and *BLM*	Analysis of additional 438 BC families (screening of all *FANCC* and *BLM* exons), 957 BC families (screening of *FANCC* mutation hotspot) and 464 healthy controls
	Kiiski et al., 2014 [47]	WES	Finland	24 individuals from 11 BC families, negative for BRCA1/2	*FANCM*	Case-control analysis in 3166 BC patients, 569 OC patients, and 2090 healthy controls
	Park et al., 2014 [48]	WES	More Countries	89 BC patients from 47 families	*RINT1*	Analysis of additional 798 BC/OC families; case-control study in 1313 BC cases and 1123 healthy controls
	Cybulski et al., 2015 [49]	WES	Poland, Canada	144 Polish and 51 French-Canadian high-risk BC patients, negative for BRCA1/2, CHEK2, NBN, PALB2 founder mutations	*RECQL*	Case-control analysis of selected mutations in 1013 BC cases and 7136 healthy controls in Canadian population, and 13,136 BC cases and 4702 healthy controls in Polish population. Segregation analysis
	Sun et al., 2015 [50]	WES	China	9 early-onset familial BC patients, negative for BRCA1/2	*RECQL*	Case-control study in 439 familial BC cases and 1588 healthy controls. Functional studies for missense variants
	Maatta et al., 2015 [51]	WES	Finland	37 individuals from 13 high-risk HBOC families	18 candidate variants in DNA damage response (DDR) pathway genes. In particular, variants in *ATM*, *MYC*, *PLAU*, *RAD1* and *RRM2B*	Case-control analysis in 129 HBOC patients and 989 healthy controls. Analysis of 31 breast tumours. Two variants also validated in 49 male BC patients and 909 male healthy controls
	Tavera-Tapia et al., 2017 [52]	WES	Spain	two BC patients from a non-BRCA family	*ATM*	Analysis of the *ATM* c.5441delT mutation in 1477 HBOC families and 589 healthy controls; NGS panel for *ATM* mutational screening in 392 HBOC families and 350 patients affected with diseases different from BC
	Hamdi et al., 2018 [53]	WES	Tunisia	8 individuals from 7 BC families, negative for BRCA1/2 (analysis focused on one family)	12 relevant high-risk variants in *HSD3B1*, *CFTR*, *PBK*, *ITIH2*, *MMS19*, *PABPC3*, *PPL*, *DNAH3*, *LRRC29*, *CALCOCO2*, *ZNF677* and *RASSF2* genes. 4 new breast cancer candidate genes (*MMS19*, *DNAH3*, *POLK*, *KATB6*)	
	Lu et al., 2019 [54]	WES	USA	11.416 HBOC patients and 3.988 controls	*ATM*, *CHEK2*, *PALB2*, *MSH6* in Breast cancer; *MSH6*, *RAD51C*, *TP53*, *ATM* in Ovarian cancer	
	Girard et al., 2019 [55]	WES	France	100 familial BC patients, negative for BRCA1/2	Selection of 77 genes plus 36 candidate BC-related genes (N = 113 genes) for validation analysis	Case-control study: Targeted sequencing of 113 DNA repairing genes in 1207 BC cases and 1199 healthy controls. Significant association between *PALB2*, *ATM*, *CHEK2*, *FANCI*, *MAST1*, *POLH* and *RTEL1* mutations and BC risk
	Weitzel et al., 2019 [56]	WES (focused on 12 known and candidate cancer susceptibility genes)	Hispanic women	1.054 familial BC patients, negative for BRCA1/2	*CHEK2*, *PALB2*, *ATM*, *TP53*, *BRIP1*, *CHD1*, *NF1*	Case-control analysis using 1189 healthy controls and data from Exome Aggregation Consortium (ExAC) database
	Glentis et al., 2019 [57]	WES	Greece	52 individuals from 17 HBOC families	*BARD1*, *MEN1*, *MDM1*, *NBEAL1*. Missense variants in *SETBP1* and *C7orf34*	Case-control analysis using 51 Canadian HBOC patients of European ancestry (FBRCAX), 512 Canadian BC patients (CHUM-BC) and 1940 healthy controls (CARTaGENE), as well as data from The Cancer Genome Atlas (TCGA) and Exome Aggregation Consortium (ExAC) databases
WGS studies	Nones et al., 2019 [58]	WGS (germline and tumor)	Australia	78 matched germline and tumour samples from individuals with and without mutations in BRCA1/2	*BRCA1/2*, *PALB2*, *MUTYH*, *TP53*, *ATM*, *CHEK2*	
